# The Prevalence of Systemic Venous Congestion Post Kidney Transplant Detected by Point of Care Ultrasound (POCUS)

**DOI:** 10.24908/pocusj.v10i01.18260

**Published:** 2025-04-15

**Authors:** Santiago Beltramino, Agustín Manchado Bruno, Damián Fernández, Javier Walther, Gustavo Werber

**Affiliations:** 1 Intensive Care Unit, Instituto de Trasplante y Alta Complejidad. Buenos Aires, ARG.; 2 Kidney Transplant Unit, Instituto de Trasplante y Alta Complejidad. Buenos Aires, ARG

**Keywords:** Venous congestion, delayed graft function, kidney transplant, POCUS

## Abstract

Systemic venous congestion is a known cause of acute kidney injury (AKI), but its presence in kidney transplant patients has not been previously described in the literature. The objective of this study was to determine the prevalence of systemic venous congestion in recent kidney transplant recipients. We conducted a prospective, longitudinal, descriptive study including 30 adult patients during the first week post-renal transplant at the Instituto de Trasplante y Alta Complejidad in Buenos Aires, Argentina. Venous congestion was detected in 53% of patients (16/30), but only 13.3% (4/30) presented moderate to severe congestion. Pulmonary congestion was more frequent: 70% (21/30) of the patients presented some degree of pulmonary congestion, and 30% (9/30) had moderate or severe congestion. In the venous congestion group, 75% of patients developed delayed graft function (DGF) compared to 57% in the non-congestion group, although this difference was not statistically significant (p<0.3). Body weight and physical examination—two commonly used methods to guide decisions on dialysis initiation and fluid management—were found to be unreliable for assessing the true volume status. In conclusion, venous congestion was observed during the first week following renal transplantation; however, moderate to severe congestion was uncommon, affecting only 13.3% of patients. While DGF was more frequently observed in patients with congestion, a statistically significant association could not be established. Further studies with larger sample sizes are needed to better evaluate this potential relationship.

## Introduction

Postoperative management of kidney transplantation, like other surgical interventions, includes fluid replacement to ensure adequate graft perfusion. However, excessive fluid administration is associated with deleterious effects, such as hypervolemia, which can lead to complications like heart failure, pulmonary edema, and impaired renal function [1]. In recent decades, fluid overload has played a major role as the triggering and aggravating factor of acute kidney injury (AKI) in critically ill patients [2]. The recommendations for post-transplant management underline the importance of preventing fluid overload; however, no clear management guidelines have been designed [3].

Point of care ultrasound (POCUS) allows hemodynamic evaluation in the critical care, nephrology, or internal medicine units. The sensitivity and specificity of POCUS are higher than those of chest X-ray and physical examination combined [4–6]. Additionally, it is used to evaluate the venous system in a non-invasive manner[7,8].

Alterations in the intra-renal venous flow as obtained by Doppler in patients with heart failure (HF) have shown an independent prognostic value for death and hospitalization at one year.9 In 2020, Beaubien-Souligny et al. developed an ultrasound-guided classification system for venous congestion, called VExUS, in the context of postoperative cardiac surgery management as a predictor for AKI. In summary, when the diameter of the inferior vena cava (IVC) is ≥2 cm with less than 50% respiratory variability, three stages of systemic venous congestion are defined based on the analysis of the pulsed Doppler waveform in the hepatic vein (HV), portal vein (PV), and intrarenal vein [10]. In this study, 96% of the patients with severe venous congestion and 87% with moderate congestion, as determined by VExUS, developed AKI. Here, the IVC diameter performed poorly as an isolated method (40% specificity), while the central venous pressure (CVP) >12 mmHg (80%) performed better.

Delayed graft function (DGF) is found in about 20-70% of transplant patients receiving a kidney from a cadaveric donor and could be associated with a higher risk of rejection and a lower graft survival rate [11–13]. It is usually a multifactorial cause. The aim of this study was to describe the prevalence of systemic venous congestion after kidney transplant. The secondary objective was to evaluate the potential association with DGF, pulmonary congestion, postoperative complications, and the effectiveness of physical examination and body weight to determine blood volume status.

## Materials and Methods

An observational study, with data collected prospectively, was conducted at the Instituto de Trasplante y Alta Complejidad (ITAC) in Buenos Aires, Argentina. Thirty adult patients with end-stage kidney failure receiving a kidney from either a cadaveric or living donor were evaluated during the short-term post-transplant period, between September 2020 and February 2021. Mindray (Shenzhen, China) equipment was used with a 2.5-5 MHz convex transducer. All physicians performing this evaluation had more than 30 hours of training in POCUS in critical care. The images were analyzed by three external ultrasound experts. The physicians performing the ultrasound studies did not take any therapeutic decisions. Additionally, the attending physicians did not have any information about the hemodynamic evaluation with POCUS.

Recipients older than 18 years of age who signed the informed consent were included. Recipients of dual organ transplants were excluded. The study was approved by the teaching and research committee and the bioethics committee.

Both epidemiological and clinical data from the post-op hospitalization period were collected. DGF and cold ischemia time were recorded. DGF was defined as the need for hemodialysis during the first week after transplantation, with the day of the transplant considered as Day 0. POCUS assessments were performed on days 1, 3, 5, and 7 after transplant. If the patient had an indication for dialysis, the studies were performed before starting dialysis.

The venous congestion evaluation was conducted according to the VExUS protocol designed by Beaubien-Souligny et al.10 The IVC was measured approximately 1 cm from the right atrium, and respiratory variability was recorded. A qualitative analysis of the flow pattern of the PV, right or middle HV, and interlobar vein (IRVD) of the graft was obtained by pulse Doppler. Based on the findings, patients were classified according to the degree of congestion as normovolemic and with mild, moderate, or severe congestion. A hypovolemia category was added if the IVC was under 0.5 cm.

*Patients were classified as follows:*

- Grade -1: IVC <0.5 cm (hypovolemic)
- Grade 0: IVC <2 cm or >2 cm with collapse >50% (normal)
- Grade 1: IVC> 2 cm and under 50% variability with no other abnormalities or mild abnormalities (mild congestion)
- Grade 2: IVC> 2 cm + 1 severe abnormality (moderate congestion)
- Grade 3: IVC> 2 cm + 2 severe abnormalities (severe congestion)

Pulmonary congestion was assessed by lung POCUS in 16 areas, 8 in each hemithorax: The second and fourth intercostal space at the parasternal level, anterior axillary level, posterior axillary, and dorsal regions were considered. The presence of more than three B-lines was indicative of pulmonary congestion in two or more adjacent areas in both hemithoraces. The presence of pleural effusion was quantified using the distance between the pleural and pulmonary lines on expiration. It was classified as trivial (<2 mm), mild (2-15 mm), or moderate-severe (>15 mm) [12,13].

*Pulmonary congestion was classified as follows* (bilateral, diffuse, and symmetrical involvement) [14]

- Grade 0 (normal) = <4 fields.
- Grade 1 (mild) = 4-7 and without pleural or trivial effusion.
- Grade 2 (moderate) = 8-11 or 4-7 + moderate-severe pleural effusion.
- Grade 3 (severe) = >12 fields or >8 fields with moderate-severe pleural effusion.

On the day of the transplant, all patients underwent a chest computed tomography (CT) scan as part of the pre-transplant protocol. This allowed for the identification of interstitial abnormalities or parenchymal disease that could account for abnormal findings on lung ultrasound unrelated to pulmonary congestion, thereby enhancing the specificity of the method.

## Statistical analysis

All quantitative variables were expressed as mean and interquartile range (IQR), and qualitative variables as absolute and relative frequencies. For comparisons of continuous variables (e.g., percentage of weight gain) between groups (e.g., congestive vs. non-congestive), we used the Mann-Whitney U test. The presence of systemic venous congestion and its severity were treated as qualitative variables, and their relationship with other qualitative variables of interest was evaluated using the chi-square test. The agreement between systemic venous congestion (according to the VExUS score) or pulmonary congestion (by lung POCUS) and clinical examination was determined with the kappa coefficient and 95% confidence intervals. All analyses were performed using R version 4.1.0.

## Results

A total of 30 patients receiving post-kidney transplant care were included in the study. Of these, 13 (43.3%) were female. The mean age was 41.5 years (IQR 32–58.25 years), and the average Body Mass Index (BMI) was 25.61. The underlying cause of end-stage kidney disease and the need for dialysis were identified in 70% of the patients (Table 1). Prior to transplantation, 28 patients exhibited preserved cardiac function (ejection fraction (EF) >45%), while 2 patients had an EF <45%.

**Table 1.** Recipients and donors

**Recipients**		
**Age (M /IQR)**	41.5 (32-58.5)	
**Gender Female**	43.30%	
**Residual urine output**	46%	
**BMI**	25 (24-28)	
**LVEF >45%**	93.3%	
**DGF**	66.70%	
**Donors**		
	**Cadaveric donors (n=24)**	**Living donors (n=6)**
**Age**	47.85 (16.65)	56 (42.5-57)
**Cold ischemia time (M/IQR)**	20 (15-23)	-
**Donor's creatinine level (m /IQR)**	1 (0.75-2.72)	0.9 (0.8-1)
**NA**	10 (50%)	-
**NA dose (mcg/kg/min)**	0.1 (IQR 0.1-0.2)	-
**BMI**	25 (24.5–26.25)	28.5 (26.75-31.25)

**Abbreviations: BMI**: Body Mass Index; **DGF**: Delayed Graft Function; **LVEF**: Left Ventricular Ejection Fraction; **NA**: Noradrenaline

During the first week after transplantation, 111 POCUS exams were performed, revealing venous congestion in 24 of them (22%), with only 6 (5%) classified as moderate or severe. Overall, 16 out of 30 patients (53.3%) studied exhibited venous congestion at some point during the first week. In most cases, the congestion was mild; only 4 patients (13%) showed moderate (2) or severe (2) congestion. In this period, only two patients met the hypovolemia criteria. Of all patients who developed systemic venous congestion at some point in time during the postoperative period, 12/16 (75%) developed DGF vs. 8/14 (57%) in the non-congestive group. In spite of these differences the values in this sample were not statistically significant (p= <0.3).

A total of 110 lung POCUS examinations were performed, of which 34 (31%) revealed signs of congestion. Among those, 11 (10%) were classified as moderate or severe. Overall, 21 out of 30 patients (70%) had some degree of congestion, and 9 (30%) presented moderate to severe congestion. Similar to the venous congestion group, DGF was more frequent in the pulmonary congestion group (72 % vs. 44%), but without significant differences (p= 0.39) (Tables 2 and 3).

**Table 2.** Systemic venous congestion

Venous congestion	Day 1	Day 3	Day 5	Day 7
**Hypovolemia**	-	-	1 (3.6%)	1 (4.3%)
**Normal**	26 (86.7%)	21 (70%)	20 (71.4%)	18 (78.3)
**Mild**	3 (10%)	7 (23.3)	5 (17.9%)	3 (13%)
**Moderate**	-	1 (3.3%)	1 (3.6%)	-
**Severe**	1 (3.3%)	1 (3.3%)	1 (3.6%)	1 (4.3%)

**Table 3.** Pulmonary congestion

Pulmonary congestion	Day 1	Day 3	Day 5	Day 7
**Normal**	22 (73%)	21 (70%)	18 (64.3%)	17 (73.9%)
**Mild**	7 (23.37%)	5 (16.7%)	5 (17.9%)	6 (26.1%)
**Moderate**	2 (6.7%)	2 (6.7%)	4 (14.3%)	-
**Severe**	-	2 (6.7%)	1 (3.6%)	-

Based on the analysis during the post-op period, no agreement was seen between the findings of the global physical examination and the POCUS assessment; both for the evaluation of the systemic venous circuit (kappa 0.122 CI 95% -0.033 to 0.277) and the pulmonary circuit (kappa 0.138 CI 95% -0.054 to 0.33). The clinical assessment was accurate in only one of the four cases of moderate-severe venous congestion and in no patients with moderate-severe pulmonary congestion. Weight gain was observed in all the cases in the post-op period. The percentage of weight gain in relation to the baseline weight was 7.83%, 8.47%, 8.1% and 7.1% for post-op days 1, 3, 5 and 7, respectively (See tables). This increase was higher in patients with POCUS echographic data indicating systemic venous or pulmonary congestion (Table 4).

**Table 4.** Weight gain in postoperative

	**Normal VEXUS**			**Congestive Vexus**			
	n	Mean (%)	CI95	n	Mean (%)	CI95	
**Day 1**	3	5	−2.95 to 12.45	3	10.66	4.92 to 16.04	**p 0.30**
**Day 3**	20	7.89	6.36 to 9.41	8	9.91	4.30 to 15.52	**p <0.01**
**Day 5**	18	7.45	5.04 to 9.86	7	9.76	6.52 to 12.99	**p <0.01**
**Day 7**	17	6.48	3.4 to 9.6	4	9.73	7.04 to 12.41	**p 0.15**
	**Normal lung**			**Congestive lung**			
	n	Mean (%)	CI95	n	Mean (%)	CI95	
**Day 1**	4	5.75	1.18 to 10.32	2	12	7.57 to 16.40	**p 0.20**
**Day 3**	20	7.78	5.53 to 10.03	8	10.18	7.82 to 12.80	**p <0.01**
**Day 5**	16	6.11	4.54 to 7.67	9	11.62	8.15 to 15.42	**p <0.01**
**Day 7**	15	5.91	3.43 to 8.38	6	10.08	8.12 to 12.01	**p <0.01**

*The values represent the percentage of weight gain compared to the patient's baseline weight.

## Discussion

There is no doubt about the relevance of venous congestion in the occurrence or progression of kidney failure. However, to date it has not been described in relation to kidney transplant. Our study is the first to describe the presence of venous congestion in transplant patients using POCUS. Although slightly more than half of the patients experienced congestion at some point during their clinical course, only 24 out of 111 POCUS examinations detected congestion, with 6 cases classified as moderate or severe. It is worth noting that 4 of the 30 evaluated patients (13.3%) presented moderate or severe congestion. This is of interest since the VExUS examination allows a rapid and noninvasive evaluation of organic congestion, identifying a high risk of AKI in cases of moderate or severe congestion. Although DGF has multiple causes, it would be reasonable to expect that venous congestion may also contribute to its development in some patients. It has been known for more than 100 years that raising renal vein pressure above 10 mmHg causes oliguria [15,16]. However, it took many years for this topic to be of interest or relevance in internal medicine. In causing AKI, CVP is more important than the fall in mean arterial pressure [17,18]. Despite this, CVP does not always correlate with the venous congestion of the organs. In addition to blood volume, venous pressure is also influenced by neurohormonal mechanisms, the compliance of the venous system and the specific characteristics of each organ. The kidney, being an encapsulated organ, is less compliant and with increases in intrarenal venous pressure which causes edema and increased interstitial pressure, arterial perfusion and glomerular filtration are affected. Therefore, it has not been possible to establish a CVP target value to prevent DGF [19–21]. Although the differences were not significant, there was a tendency to develop DGF in the group with venous congestion vs. the group without venous congestion. Studies with a larger number of patients will be necessary to determine whether there is an association between venous congestion and DGF.

**Figure 1.**
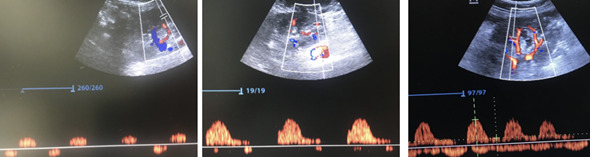
Case 2. Evolution of Doppler interlobar point of care ultrasound (POCUS), arterial flow over the baseline and venous flow under the baseline. In A, RRI 1 with venous flow alone in the diastole (maximum venous congestion). In B after a negative balance of 5 kg on dialysis a better arterial flow and moderate venous congestion are seen (flow in both times). In C, RRI 0.75 with normal venous flow after a negative balance of 10 kg on dialysis.

Of patients with severe congestion, one had received a kidney from a living donor with no history of cardiac disease. Severe venous congestion was found on the first day, with both the PV and HV involved without impairment of IRVD. The patient developed polyuria and both venous ultrasound evaluation and renal function normalized in the following days. The second case was a patient with a history of heart failure with reduced systolic function (EF 38%) who received a kidney from a cadaveric donor. On the second evaluation (Day 3), the patient developed severe venous congestion with HV, PV and IRVD involvement sustained for some days, with mild pulmonary congestion. During the first week, his urine output ranged between 1000-2000 mL/day and required only one hemodialysis session due to high uremia. The clinical assessment by the attending physicians always showed normovolemia and the weight gain was 10% as compared to the initial weight. The patient developed heart failure on Day 10 after transplant with signs of low cardiac output. He needed hemodialysis, vasopressors and respiratory support. He even tolerated 5% less under the dry weight reported on admission (Figure 1).

Pulmonary congestion was more frequent than venous congestion, with 34 out of 110 lung POCUS examinations performed (31%), of which 11 (10%) were classified as moderate or severe. Seventy percent of the patients showed congestion at some point during the evaluation, and 42.8 % of these had moderate or severe congestion. Similar to the venous congestion group, patients with pulmonary congestion exhibited a higher tendency to develop DGF compared to those without pulmonary congestion (72% vs. 44%, respectively). Regarding lung POCUS, we found no previous studies that specifically focus on its application in the context of renal transplantation.

In our study, the physical examination did not correlate with the POCUS evaluation in the majority of patients. Clinical assessment was accurate in only one out of four patients with significant venous congestion, and in none of the cases with moderate or severe pulmonary congestion. POCUS has proven to be a valuable tool for diagnosing the causes of dyspnea with greater accuracy than physical examination and chest X-rays combined. In patients with CHF, it is instrumental in guiding diuretic therapy to reduce hospital length of stay and rehospitalization rates [22–25]. Additionally, it plays a critical role in managing fluid balance during dialysis, helping to optimize dry weight and arterial pressure [26]. In a multicenter trial involving 392 patients on chronic hemodialysis, nearly half presented moderate to severe pulmonary congestion, with 71% being asymptomatic or exhibiting only mild CHF symptoms [27]. However, no studies have specifically explored the use of pulmonary ultrasound in the context of renal transplantation, and its application in this field remains limited. Effective blood volume management and the prevention of fluid overload are essential to reducing the risk and frequency of dialysis sessions, as fluid overload has been shown to negatively impact kidney graft outcomes [28].

Additionally, physical examination fails to identify critically ill patients with low cardiac output [29]. In our study, two patients exhibited signs of hypovolemia (IVC <0.5 cm) but were clinically assessed as normovolemic.

In daily practice, body weight is usually used as a tool to make decisions about hemodialysis and determine the volume to be removed during the post-op period; a slight weight increase is considered normal. This is related to hemodynamic changes occurring after grafting with a significant decrease of CVP in spite of aggressive fluid resuscitation with crystalloids. This decrease occurs after declamping and is associated with graft reperfusion and the generation of free radicals and cytokines causing venous vasodilation and capillary leak [30]. This could explain why pulmonary congestion was more frequent than venous congestion. Thus, as expected, weight gain was consistent in this series of patients but did not always reflect venous or pulmonary congestion. Volume overload is not synonymous with congestion. Therefore, body weight should not be used as an isolated method to understand a patient's condition.

In conclusion, we know that venous congestion is a very important mechanism for the development or progression of AKI. This is the first study showing the occurrence of venous congestion in the short term after kidney transplant and more frequently pulmonary congestion. Although no significant difference was seen, the incidence of DGF was higher among patients with congestion, venous and pulmonary. It will be important to conduct new studies with a larger number of patients to evaluate this possible association. It is important to note that both the physical examination and body weight, two parameters commonly used to decide the need for dialysis and fluid balance, do not correlate well with blood volume status. The incorporation of multi-organ ultrasound evaluation allows obtaining a greater accuracy in clinical assessment and thus improves clinical decision.
